# Editorial: Growth and development in horticultural crops: mechanisms, regulation, and innovation

**DOI:** 10.3389/fpls.2026.1947377

**Published:** 2026-09-09

**Authors:** Wei Qian, Ainong Shi, Liwang Liu

**Affiliations:** 1 State Key Laboratory of Vegetable Biobreeding, Institute of Vegetables and Flowers, Chinese Academy of Agricultural Sciences, Beijing, China; 2 Key Laboratory of Biology and Genetic Improvement of Horticultural Crops, Ministry of Agriculture, Beijing, China; 3 Department of Horticulture, University of Arkansas, Fayetteville, AR, United States; 4 National Key Laboratory of Crop Genetics & Germplasm Enhancement and Utilization, Key Laboratory of Horticultural Crop Biology and Genetic Improvement (East China) of MOAR, College of Horticulture, Nanjing Agricultural University, Nanjing, China

**Keywords:** disease resistance, flowering control, fruit ripening, horticultural crops, molecular regulation, plant architecture, stress tolerance

Horticultural crops, including fruits, vegetables, ornamentals and medicinal plants, are indispensable to global food security, human nutrition, and economic livelihoods. Yet their productivity and quality are increasingly jeopardized by climate change, resource scarcity, and the growing demand for sustainable production systems. Methods such as molecular biology and genomics offer opportunities to decipher how genetic, physiological, and environmental factors jointly regulate the growth performance of horticultural crops. This Research Topic brings together fourteen original research articles and one comprehensive review that collectively advance our understanding of the molecular and environmental mechanisms governing growth, development, and quality in a wide spectrum of horticultural species, including *Solanum lycopersicum*, *Spinaca oleracea*, *Asparagus officinalis*, *Prunus persica*, *ragaria × ananassa*, *Ziziphus jujuba* var. *spinosa*, *Pyrus bretschneideri*, *Spathiphyllum kochii*, *Cucumis melo*, *Cymbidium*, *Lycium barbarum*, and *Ananas comosus*.

## Fruit ripening and quality

Fruit ripening is a tightly regulated developmental transition with direct implications for postharvest shelf life, nutritional value, and consumer acceptance. Three studies illuminate novel regulatory nodes in tomato and strawberry. Sun et al. demonstrate that the receptor-like kinase SlRLK-like acts as a positive regulator of tomato ripening by interacting with SlSWEET sugar transporters to enhance sugar accumulation, while also engaging ethylene and lycopene biosynthesis proteins (SlACS2, SlSAMS4, SlPSY1) to promote ethylene production and pigmentation. In contrast, Li et al. identify RAR, a previously uncharacterized NAC transcription factor, as a negative modulator of climacteric ripening onset. RAR is highly expressed at the mature green stage and directly represses *ACS2* expression, forming a feedback loop with ethylene that prevents premature ripening. Together, these studies reveal that ripening initiation is governed not only by positive activators but also by repressive checkpoints; this finding has clear implications for the precise-tuning harvest timing through genetic manipulation. Extending these insights to non-climacteric fruits, Chen et al. employed integrative transcriptomics and Weighted Gene Co-expression Network Analysis (WGCNA) to compare two strawberry varieties with contrasting firmness and sweetness profiles. Their stage-resolved analysis revealed that firmness retention in ‘Monterey’ is associated with sustained expression of a NAC transcription factor (*Fxa2Dg203497*), whereas rapid softening in ‘Three Princess’ correlates with early activation of specific polygalacturonases and starch/sucrose metabolism genes. This work demonstrates that varietal differences in fruit quality arise from stage-specific transcriptional interplay between cell wall-disassembling enzymes and their regulators, providing candidate genes for breeding strawberries with optimized texture and flavor.

## Plant architecture

Shoot branching, flowering time, and plant stature are fundamental determinants of yield, ornamental value, and cultivation efficiency. Hatinoğlu et al. used CRISPR/Cas9 to mutate conserved non-coding regions upstream of *SlBRC1B*, a key inhibitor of bud outgrowth in tomato. Their findings reveal that *SlBRC1B* is under tight repressor-dependent control and that targeting these cis-regulatory elements offers a strategy for fine-tuning branching without pleiotropic effects. In addition, Lai et al. identified 39 *ABCB* auxin transporter genes in melon and showed that *CmABCB14*, *CmABCB20*, and *CmABCB21* are positively correlated with IAA concentration, suggesting they function as auxin efflux carriers that suppress axillary bud growth. Plant height, a critical trait for mechanical harvesting, is addressed by Alatawi et al. through genome-wide association study (GWAS) and genomic prediction in 307 spinach accessions. They identified ten SNPs significantly associated with height and nine candidate genes within linkage disequilibrium (LD) regions, achieving a prediction accuracy of r = 0.55 using GWAS-derived markers. This work provides a robust framework for marker-assisted breeding of spinach ideotypes suited to mechanized production.

## Flowering regulation

Flowering is a pivotal developmental transition, and two contributions explore its control from different angles. Ma et al. characterized the *PEBP* gene family in sour jujube, revealing antagonistic expression dynamics between *ZjFT* and *ZjTFL1* during fruit-bearing shoot development. Notably, they identified a SMFT-like subfamily potentially originating from horizontal gene transfer, and demonstrated that all *ZjPEBP* genes are induced by ABA treatment, with *ZjSMFT* showing a 400-fold upregulation. Furthermore, Chen et al. conducted a comprehensive review of the flowering mechanisms in pineapples, summarizing the key role of ethylene in both natural and induced flowering, as well as the practical applications of growth regulators such as ethephon and AVG for synchronizing or suppressing flowering; these findings could be applied to year-round production.

## Dormancy and abiotic stress tolerance

Perennial horticultural crops must balance growth with dormancy to survive seasonal extremes. Zhao et al. identified 48 *GH17* (β-1,3-glucanase) genes in peach and showed that many of these genes respond to ABA and GA and are differentially expressed during dormancy release. They confirmed that PpGH17–8 interacts with PpKINβ2 and PpMIEL1, suggesting that intercellular communication via plasmodesmata is a key regulatory node in bud dormancy transitions. Drought stress, a growing threat, was investigated by (Jia et al.). They characterized seven BPC transcription factors in pear and validated that *PbBPC5* is a positive regulator of drought tolerance. Silencing *PbBPC5* impairs antioxidant defenses, leading to increased reactive oxygen species (ROS) accumulation and cellular damage, thereby establishing BPC transcription factors as promising targets for enhancing drought tolerance.

## Disease resistance and the power of pan-genomics

Two studies utilized comparative and pan-genomic approaches to dissect disease resistance. Sun et al. compared NLR gene families in cultivated asparagus and its two wild relatives, revealing a marked contraction of NLR genes from wild species (63) to cultivated *A. officinalis* (27). Furthermore, the remaining NLR genes exhibited reduced capacity upon pathogen infection, which is most likely attributable to artificial selection for yield and quality. She et al. constructed a pan-NLRome from 19 spinach assemblies and developed a novel pipeline integrating pan-NLRome with *K-mer*-based GWAS. This approach directly pinpointed the candidate gene for the downy mildew resistance locus *RPF1*, whereas single-genome methods identified four candidates requiring further validation. This pipeline offers a powerful strategy for rapid resistance gene cloning across multiple crops.

## Ornamental traits and reproductive biology

Beyond food crops, this Research Topic also covers ornamental and medicinal plants. Zhao et al. identified 20 structural genes in the anthocyanin biosynthesis pathway of *Cymbidium haematodes*, showing that *CsF3’H2* and *CsF3’H3* exhibit high expression levels in the red petals and are key genes. Wang et al. characterized 213 RING finger genes in goji berry and identified LbSBP1 as a self-incompatibility-associated protein that interacts with multiple pollen S-determinants, providing a foundation for breeding self-compatible varieties. Finally, Lin et al. constructed the first full-length reference transcriptome for *Spathiphyllum kochii* and identified *SpRCC1* as a positive regulator of growth, which is is significantly down-regulated in dwarfing mutants. Overexpression of this gene in *Arabidopsis thaliana* increases leaf area, a finding of significant value for ornamental plant breeding.

Collectively, the articles in this Research Topic reveal a unifying principle: developmental transitions and stress responses in horticultural crops are governed by a dynamic equilibrium between positive activators and repressive checkpoints, with hormone signaling (ethylene, auxin, ABA, and GA) serving as a central hub that integrates internal genetic programs with environmental cues. Notably, the same regulatory modules—such as NAC transcription factors, receptor-like kinases, and auxin transporters—are repeatedly co-opted across different species and traits, underscoring a deep mechanistic conservation that transcends individual case studies. However, current approaches have clear limitations: they still largely rely on single reference genomes that miss structural variations, focus predominantly on coding regions at the expense of distal cis-regulatory elements, and often leave a gap between controlled-environment functional validation and field performance due to unaccounted genotype-by-environment (G×E) interactions.

To bridge this gap, we advocate for a framework that explicitly models how genomic variation provides the genetic toolkit, molecular regulation dictates its spatiotemporal deployment, and environmental factors modulate these circuits via stress-responsive elements. Translating these findings into sustainable breeding practices will require a multi-directional effort: (1) moving from single reference to pan-genome and graph-based genomes to capture structural variations; (2) expanding from coding regions to regulatory genomics, including promoters, enhancers, and non-coding RNAs; the CRISPR/Cas9 editing of SlBRC1B cis-regulatory regions in tomato showcased here provides a compelling blueprint for fine-tuning plant architecture with reduced off-target effects. (3) integrating genotype-by-environment (G×E) interactions and phenotypic plasticity into predictive models; (4) applying artificial intelligence and deep learning to integrate multi-omics data and uncover non-linear interactions; and (5) advancing from candidate gene discovery to predictable, designable genome editing guided by pan-genomic and AI-predicted networks. We hope this Research Topic stimulates further research that bridges laboratory insights with field applications, ultimately contributing to more resilient and sustainable horticultural systems.

